# Enhancing English reading skills and self-regulated learning through online collaborative flipped classroom: a comparative study

**DOI:** 10.3389/fpsyg.2023.1255389

**Published:** 2023-10-16

**Authors:** Ying Wang

**Affiliations:** Department of Foreign Language, Liaocheng University Dongchang College, Liaocheng, Shandong, China

**Keywords:** online collaborative flipped classroom, English reading skills, self-regulated learning, online learning behaviors, EFL students

## Abstract

**Introduction:**

This research investigates the effectiveness of an online collaborative flipped classroom approach in enhancing English reading skills and self-regulated learning among Chinese English learners.

**Methods:**

A total of 71 participants were divided into three instructional groups: traditional instruction (TI) group (*n* = 24), flipped instruction (FI) group (*n* = 22), and online flipped instruction (OFI) group (*n* = 25). The participants’ reading comprehension ability was assessed using the reading section of the IELTS exam. Self-Regulated Learning (SRL) strategy use was evaluated using a questionnaire, and weekly online quizzes assessed participants’ understanding of course materials. Online learning behaviors were examined by considering online log-on times. The instruction period lasted for 12 weeks, with pre-tests and post-tests conducted to measure progress.

**Results:**

The results indicated that both the FI and OFI groups outperformed the TI group in terms of reading comprehension and self-regulated learning. Furthermore, the OFI students demonstrated superior online learning behaviors and objective performances compared to the FI students.

**Discussion:**

These findings suggest that the integration of flipped and online instruction methods holds promise for improving English reading skills and enhancing self-regulated learning among Chinese English learners.

## Introduction

Online learning has emerged as an adaptable, accessible, and efficient avenue for second language (L2) acquisition, enabling learners to assume an active role in their language learning journey (Lin et al., 2017; Basilaia and Kvavadze, 2020; Subedi et al., 2020; Pokhrel and Chhetri, 2021). In fact, the surge of online learning and digitalized education has sparked a transformative shift in the educational landscape, ushering in an era of digital transformation within this domain (Fisher, 2006; García-Morales et al., 2021; Zarifis and Efthymiou, 2022; Fathi et al., 2023; Widayanti and Meria, 2023). Amidst this evolving educational paradigm, a prominent innovative strategy has garnered widespread recognition for its student-centered ethos–the flipped classroom. This approach integrates various pedagogical elements, encompassing cooperative and collaborative learning, peer-based interactions, problem-solving techniques, and dynamic learning methods, effectively crafting an engaging online learning milieu (Slavin, 1991; Topping and Ehly, 1998; Michael, 2006; Bergmann and Sams, 2014; Hung, 2017; Fathi et al., 2021). Through this multifaceted approach, the flipped classroom leverages technology to optimize learning experiences and elevate learner engagement.

Flipped classrooms, a pedagogical approach gaining prominence in the field of English as a foreign language (EFL) instruction, are characterized by a fundamental shift in the traditional classroom paradigm. In the EFL context, a flipped classroom model involves the strategic use of digital resources to invert the conventional sequence of in-class instruction and out-of-class learning activities. This instructional approach redefines the roles of both educators and learners, fostering a more learner-centered and interactive environment. In a typical flipped EFL classroom, educators curate and deliver digital content, often in the form of video lessons, online modules, or multimedia materials, which cover core course topics and language skills. Students are provided access to these resources prior to in-person class sessions (Chen Hsieh et al., 2017). This pre-class exposure to content equips learners with foundational knowledge, enabling them to arrive at in-class sessions prepared and ready for interactive engagement. During in-person class time, the focus shifts from traditional lecturing to collaborative and application-based activities. Educators facilitate discussions, problem-solving exercises, and hands-on language tasks that encourage active participation and deeper comprehension. This approach capitalizes on the concept of “homework in the classroom” and “classwork at home, “allowing students to clarify doubts, seek clarification, and engage in peer-to-peer learning under the guidance of their instructors (Mehring, 2016).

While the flipped classroom has been recognized as a beneficial approach for enhancing EFL learners’ linguistic competence (O’Flaherty and Phillips, 2015; Shih and Huang, 2020; Turan and Akdag-Cimen, 2020), limited research exists on its impact on other variables, particularly reading comprehension–an essential skill for academic knowledge acquisition–and self-regulated learning strategies–an important tool for independent language learning (Vitta and Al-Hoorie, 2020; Fathi and Rahimi, 2022). Research suggests that an online flipped classroom model can enhance reading comprehension (Karimi and Hamzavi, 2017; Samiei and Ebadi, 2021). Reading comprehension involves constructing meaning by connecting background knowledge with textual information, making it a vital component of academic contexts (Yapp et al., 2021). Learners need to develop the ability to read independently, even in online or home settings, by engaging with texts at the word, sentence, and text levels, seeking feedback from peers through discussions, accessing resources, and reflecting on their reading practices (Jeon and Yamashita, 2014).

Flipped classrooms, a pedagogical innovation gaining traction, often leverage homework assignments focused on reading materials to cultivate learners’ autonomy, motivation, and a positive attitude toward advancing reading comprehension skills (Fulgueras and Bautista, 2020). Additionally, an intrinsic link exists between the flipped classroom model and the cultivation of self-regulated learning (SRL) strategies. Within this paradigm, learners are entrusted with the responsibility of not only acquiring and organizing information but also actively engaging in processes such as monitoring, reflection, and evaluation of their own learning practices (Lai and Hwang, 2016; Theobald, 2021). Crucially, flipped EFL classrooms prioritize the learner’s autonomy and self-regulated learning. Learners are encouraged to take ownership of their learning process, make informed decisions regarding their study pace, and employ self-regulation strategies to enhance their language acquisition process (Lai and Hwang, 2016). This instructional method harnesses technology to create a dynamic and adaptable learning ecosystem that empowers students to assume agency over their language learning.

The inclusion of self-regulated learning as a variable of interest alongside reading comprehension is guided by the understanding that these two aspects might be intertwined in a symbiotic relationship. Self-regulated learning encompasses a spectrum of behaviors, motivations, and metacognitive functions, all of which converge as students plan learning tasks, set attainable goals, track their progress, and engage in thoughtful reflection on their learning journey (Nilson, 2023). The strategic employment of self-regulated learning strategies, especially within the context of online collaborative flipped classrooms, is postulated to synergistically enhance reading comprehension abilities. This study endeavors to unravel the interplay between self-regulated learning and reading comprehension, shedding light on how the deliberate cultivation of metacognitive strategies through the flipped classroom model can potentially influence learners’ abilities to comprehend and engage with English text.

Despite separate investigations on the flipped classroom, reading comprehension, and self-regulated learning strategies, further research is needed to understand how online flipped classrooms can influence reading comprehension and self-regulated strategies. To fill this research gap, this study aims to compare the effects of online flipped instruction and traditional flipped instruction on L2 reading comprehension and self-regulated learning among Chinese EFL learners. Additionally, the study seeks to explore differences in online learning behaviors between the two instructional groups. By saturating and confirming the existing literature and generating context-based findings, this study contributes to the expanding body of research on the effectiveness of the flipped classroom model in L2 instruction. Moreover, it provides valuable insights into the impact of flipped instruction on self-regulated learning in an online setting. The findings of this study may have practical implications for language teachers and curriculum developers interested in incorporating the flipped classroom model into their language instruction.

## Literature review

### Flipped classroom

The numerous contributions of digital learning to motivate students and make students active language learners were due to its approachability, convenience, collaboration, and proximity of digital devices that could enhance autonomy and add variations to the learning process (Prensky, 2005; Murdock and Williams, 2011; Zarifis and Efthymiou, 2022). The same was advocated in Asian countries since students widely used technological features to communicate through text, video calls, and other features that could help them interact and engage (Sweeny, 2010). Enhancing student-centered approaches in the online environment, teachers can employ flipped classroom model (FCM) to change traditional class activities. FCM brings rich chances for learners, adds flexibility and adaptability (Bergmann and Sams, 2014; Shih and Huang, 2020), and offers practical tasks during class. Insights have arisen from diverse fields, spanning social sciences (Wanner and Palmer, 2015; Lee and Wallace, 2018), engineering (Karabulut-Ilgu et al., 2018), and education (Zainuddin and Attaran, 2016; Sommer and Ritzhaupt, 2018), all of which increasingly advocate the effectiveness of the flipped classroom in enhancing learners’ educational outcomes (Çakıroğlu and Öztürk, 2017; Liu et al., 2019). Furthermore, several defining characteristics have been proposed for the flipped classroom, including interactive learning (Crouch et al., 2007), real-time engagement (Novak, 2011), inverted instruction (Davis, 2013), and the flipped learning model (Bergmann and Sams, 2014).

Participating in online flipped classrooms might empower EFL learners to cultivate autonomy in their decision-making and actions, fostering a sense of ownership and control over their reading experiences (Mehring, 2016; Fulgueras and Bautista, 2020). This newfound autonomy motivates learners to proactively adapt and refine their reading strategies to meet the demands of comprehension. Moreover, learners develop a positive attitude toward the challenges encountered during the reading process, embracing them as opportunities for growth and deeper understanding (Jia et al., 2023). Furthermore, online flipped classrooms offer EFL learners avenues for improving their vocabulary and grammatical knowledge (Turan and Akdag-Cimen, 2020). Prior to, during, or after reading a text, learners can leverage various techniques to enhance their language proficiency (Jiang et al., 2022). These include consulting dictionaries to clarify unfamiliar words, utilizing contextual clues to predict and deduce meanings, engaging in discussions with peers to elicit insights, and employing effective organizational strategies such as rehearsal, rereading, and summarization (Mohammaddokht and Fathi, 2022). By employing a range of cognitive and metacognitive strategies, learners optimize their reading experience and foster a deeper understanding of the text (Kintsch, 2012; Fischer and Yang, 2022). Through the interactive and collaborative nature of online flipped classrooms, EFL learners engage in a multifaceted approach to reading. They not only improve their linguistic competencies but also develop critical thinking skills, cultivate effective study habits, and foster a reflective stance toward their reading practices (Fulgueras and Bautista, 2020; Samiei and Ebadi, 2021). This comprehensive approach to reading instruction nurtures learners’ confidence, self-efficacy, and motivation, positioning them for success in their language learning journey.

The flipped classroom can indirectly use technology, mobile, and computers outside of the classroom by watching videos of lectures, working with multimedia with other peers to gain knowledge and information (Kiernan and Aizawa, 2004; Stockwell, 2013; Amer, 2014), and have more considerable engagement in problem-solving, knowledge-sharing, and information-exchange communicative activities which are meaningful, along with personalized feedback in the classroom (Kim et al., 2017; Zarrinabadi and Ebrahimi, 2018). The benefits of experiencing flipped classroom can be positive perceptions of being actively involved, having more engagement, enhancing autonomy and critical thinking (Critz and Knight, 2013), and reaching greater achievement (Butt, 2014). The empirical findings also add novel findings to the literature. Accordingly, after investigating 66 pre-service English language teachers, Gok et al. (2021) found that there could be a considerable decline in FL classroom anxiety and reading anxiety during the flipped classroom. In another study, Jiang et al. (2021) revealed that learners’ demeanor and others’ assistance could moderate the significance of preparation to be motivated and involved in an online flipped classroom.

Numerous investigations have explored the influence of flipped classroom methodologies on the reading proficiencies of EFL students and associated variables. In their study, Mohammaddokht and Fathi (2022) noted that flipped instruction produced substantial enhancements in EFL reading capabilities while concurrently alleviating reading-related apprehension. These findings imply the potential utility of flipped instruction in the context of EFL reading courses. Correspondingly, Fulgueras and Bautista (2020) scrutinized the repercussions of flipped classrooms on the development of critical thinking abilities and reading comprehension among senior high school ESL learners in the Philippines. The outcomes indicated advancements in critical thinking and reading comprehension proficiencies for both the flipped and conventional lecture-discussion pedagogies. However, the flipped learning approach unequivocally outperformed its traditional counterpart, underscoring its effectiveness in fortifying these competencies.

Examining student viewpoints on the implementation of flipped classrooms in EFL reading classes during the Covid-19 pandemic, Nursyahdiyah et al. (2022) conducted a case study that unveiled the efficacy of the flipped classroom strategy in enhancing the caliber of EFL learning. Furthermore, it fostered greater autonomy among students in their learning endeavors and positively influenced the role of technology in the realm of education. Yulian’s research (2021) established that the adoption of the flipped classroom paradigm led to enhancements in critical thinking skills pertinent to critical reading. Students expressed favorable perceptions of this approach, placing emphasis on self-guided learning as a principal advantage. Likewise, Maharsi et al. (2021) scrutinized the integration of the flipped classroom approach within an EFL private university in Indonesia. The findings underscored that conventional classrooms exhibited augmented post-test scores in comparison to their flipped classroom counterparts, potentially attributed to teacher-centric instructional methods and technology-related variables. Nevertheless, a significant portion of students perceived flipped classrooms as catalysts for self-reliant and dynamic learning experiences, with recognition of both the merits and demerits associated with this approach.

In addition, Li et al. (2022) delved into the repercussions of employing the flipped classroom paradigm within the sphere of EFL instruction. Their inquiry strategically probed the manner in which the flipped methodology can augment the acquisition of students’ communicative competence. The outcomes of their investigation cast a revealing light upon the potential advantages associated with integrating the flipped pedagogical approach into the domain of EFL instruction, thus furnishing insights into the realms of inventive language learning methodologies. In an exploration conducted by Liu et al. (2022), salient revelations emerged regarding the efficacy of the flipped framework in amplifying both writing prowess and the utilization of metacognitive strategies. Their inquiry makes a notable contribution to the comprehension of how the flipped classroom configuration can positively influence not only writing proficiency but also the maturation of metacognitive faculties in the context of collaborative writing. Similarly, Shih and Huang (2020) centered their inquiry on the adept application of metacognitive strategies among college students in an EFL flipped classroom milieu. Through an intricate analysis of students’ deliberate utilization of metacognitive strategies, their research enriches the understanding of the intricate dynamics underlying students’ cognitive processes and strategic approaches within the flipped learning milieu.

Su Ping et al. (2020) also embarked on a scholarly exploration of the trajectory undertaken by EFL students within the framework of a flipped classroom, with a specific focus on a writing-intensive class. Hailing from the educational landscape of Malaysia, their research presents a distinctive vantage point that brings to light the array of experiences and outcomes that unfold for EFL learners immersed in a writing-centered flipped classroom setting. Engaging in a methodical appraisal, Turan and Akdag-Cimen (2020) executed a comprehensive dissection of the implementation of the flipped classroom methodology in the context of English language instruction. Their amalgamation of research findings furnishes substantial revelations into the overarching efficacy and repercussions of the flipped pedagogical approach in the domain of language learning. This synthesis substantially enriches the broader comprehension of the multi-faceted dimensions inherent in the implementation of the flipped approach. Delving into the intersection of pedagogy and technology, Jiang et al. (2021) undertake an investigative expedition into the amalgamation of automatic speech recognition technology within the structure of a flipped classroom. Their inquiry unveils the latent potential of technology to elevate the complexity of EFL learners’ oral language capabilities, effectively interweaving modern technological advancements with the foundations of the flipped learning milieu. In another study, Karjanto and Simon (2019) explored the application of the flipped classroom methodology in a Calculus course situated within a cultural context influenced by Confucian heritage. They designed a theoretical framework that integrated elements such as Bloom’s taxonomy, English-medium instruction, and the incorporation of technology. Their instructional design encompassed four distinct approaches, including variations of the flipped classroom model. The quantitative analysis yielded notable findings, revealing a significant discrepancy in examination scores, particularly evident when comparing fully-flipped instruction to single-topic flipped instruction. Furthermore, their qualitative investigations underscored positive enhancements in student engagement and interactions with instructors. Nevertheless, they also unearthed challenges linked to language, cultural factors, competition, and the adaptation to technological tools.

Taken together, these studies contribute to the literature by examining the efficacy of the flipped classroom approach in enhancing various dimensions of EFL learning, including communication, writing, metacognition, critical thinking, and oral language proficiency. Their insights resonate with the evolving landscape of language education, offering valuable guidance for educators seeking to embrace innovative pedagogical strategies to meet the diverse needs of language learners.

### Reading comprehension

Reading comprehension is a multifaceted skill that involves various cognitive processes and strategies. According to Kintsch (2012), reading comprehension entails the ability to connect existing knowledge (schema) with the information presented in the text, summarize key elements, draw appropriate conclusions, and enhance understanding by posing probing questions. It encompasses the process of constructing meaning from written texts, ranging from recognizing individual symbols and linguistic units to synthesizing and integrating information within a meaningful framework, thereby engaging higher-order thinking skills (Kendeou et al., 2014; Zhang and Zhang, 2022).

Comprehending a written text is a complex cognitive activity that relies on several interconnected factors. It necessitates the activation of prior knowledge, fluency in reading, relevant past experiences, the utilization of cognitive and metacognitive strategies, a strong grasp of lexical and grammatical knowledge, the ability to organize information, make judgments, and engage in reflective evaluation (Syatriana, 2011). Consequently, reading comprehension is recognized as a challenging skill in internationally recognized tests such as the International English Language Testing System (IELTS) and Test of English as a Foreign Language (TOEFL) (Pellegrino and Hilton, 2012; Hung, 2015). Furthermore, several other variables can influence learners’ reading comprehension abilities. These variables encompass reading types, individuals’ attitudes toward reading, the methods employed during reading activities, adaptability to different text genres, and the strategies utilized by learners to comprehend the texts effectively (Jeon and Yamashita, 2014; Zhang and Zhang, 2022). These factors interact and contribute to learners’ overall reading comprehension performance (Jeon and Yamashita, 2014). Given the complexity of reading comprehension and its significance in academic and language proficiency assessments, it is imperative to further explore various instructional procedures and their impact on learners’ comprehension abilities (Yapp et al., 2021). Also, understanding the variables that affect reading comprehension can inform instructional practices, curriculum design, and the development of effective strategies to enhance learners’ reading skills.

Reading comprehension, a challenging process that contains components, procedures, and aspects with the desire to discover great ways of accelerating it, is an integrated process of generating meanings from a reading section (Meniado, 2016). Besides, there appeared several ways to improve EFL learners’ reading comprehension, for instance, by incorporating online flipped classrooms, as supported in the literature (Öztürk and Çakıroğlu, 2021; Samiei and Ebadi, 2021; Fischer and Yang, 2022; Hasan et al., 2022; Mohammaddokht and Fathi, 2022). According to Samiei and Ebadi (2021), WebQuest-based flipped classroom significantly enhances learners’ inferential reading comprehension as revealed via the data analysis. In a similar study, Hashemifardnia et al. (2018) examined how flipped classroom affects junior high school students’ reading comprehension in EFL context. They stated that online flipped classrooms could substantially affect reading comprehension. Although the significance of flipped classrooms in enhancing reading comprehension has received limited exploration, the objective of this study is to contribute to the existing literature by investigating the impact of online flipped classrooms on the reading comprehension of EFL learners.

### Self-regulated learning

In educational psychology, Self-Regulated Learning (SRL) stands as a foundational construct, intrinsically linked with Zimmerman’s theoretical framework (Zimmerman, 2000). Zimmerman posits that human regulatory skill, or the lack thereof, holds a pivotal role in shaping our perception of personal agency, which, in turn, forms the very core of our self-concept (Zimmerman, 2000; Zimmerman and Schunk, 2001). The development of this regulatory capability, encompassing its subcomponents and functional aspects, has remained a central focus of social cognitive theory and research (Zimmerman, 2000; Zimmerman and Schunk, 2001).

Zimmerman’s comprehensive framework extends its purview to elucidate common dysfunctions observed in self-regulatory functioning, including phenomena such as biased self-monitoring, self-blaming judgments, and defensive self-reactions (Zimmerman, 2000). In seeking to provide a holistic perspective on self-regulation, Zimmerman’s framework addresses a multitude of facets. These include delving into the structural elements of self-regulatory systems, discerning the influences of social and physical environmental contexts on self-regulation, investigating dysfunctions that may arise within the realm of self-regulation, and exploring the developmental trajectory of self-regulation (Zimmerman, 2000).

Within the realm of SRL, learners engage in a multifaceted set of strategies that empower them to meticulously plan, closely monitor, and critically evaluate their learning activities (Zimmerman and Schunk, 2001). These strategies, deeply ingrained in Zimmerman’s model, serve as the scaffolding upon which learners construct their self-regulated learning processes. They assume control over their learning endeavors, establish meaningful goals, evaluate their progress, and adapt their strategies judiciously to optimize learning outcomes (Zimmerman and Schunk, 2001).

These SRL strategies seamlessly align with three distinct phases: Planning, Monitoring, and Evaluating. In the *Planning* phase, learners undertake activities that lay the groundwork for effective reading comprehension. This phase encompasses actions such as previewing reading tasks, setting clear learning objectives, and formulating goals before immersing themselves in the reading materials.

The *Monitoring* phase, on the other hand, hinges on learners’ adeptness at overseeing their reading progress and performance. Strategies like self-checking comprehension, identifying challenging sections, and making real-time adjustments during the reading process epitomize this phase.

Lastly, the *Evaluating* phase revolves around the critical assessment of the efficacy of one’s learning strategies and the attainment of learning objectives. In this phase, learners engage in reflection on their reading experiences, conduct a thorough analysis of the success of their approaches, and contemplate adjustments for future learning endeavors.

In the specific context of English reading comprehension, the application of SRL strategies assumes paramount importance. Learners can substantially enhance their reading skills by proactively employing SRL strategies that seamlessly align with Zimmerman’s model. These strategies, which traverse the planning, monitoring, and evaluating phases, enable learners to not only navigate the intricate landscape of reading comprehension effectively but also become architects of their own learning experiences. In the planning phase, learners prelude their reading journeys by engaging in activities such as previewing reading tasks and crystallizing their learning objectives. Subsequently, the monitoring phase calls for ongoing self-assessment and vigilant tracking of progress during the reading process. Finally, the evaluating phase encourages learners to engage in a comprehensive assessment of their comprehension, scrutinize the efficacy of their chosen strategies, and craft a roadmap for continued learning success.

Self-regulated learning strategies hold significant importance in empowering students to assume agency over their learning process and actively participate in their educational journey (Zimmerman, 2002). Self-regulation encompasses the development of a practical understanding of one’s own abilities, enabling students to make informed decisions and take appropriate actions to enhance their learning experiences (Zimmerman, 2000; Pajares, 2009). Students who possess a high level of self-regulation acquire the capacity to exert control over their learning processes, actively constructing meaning, establishing goals, making deliberate choices regarding the strategies they employ, and assuming leadership in directing their own learning (Zimmerman and Schunk, 2001; Pintrich, 2004). Moreover, they effectively integrate contextual and personal factors into their learning experiences, recognizing the interplay between these elements.

The utilization of self-regulated learning strategies results in increased engagement and proactive learning among students (Nilson, 2023). They develop the ability to monitor their progress, adapt their learning strategies as needed, and demonstrate perseverance in the face of challenges. This acquisition of self-regulation empowers students to become autonomous learners, capable of adjusting their approaches to different learning tasks and contexts, thereby enhancing the effectiveness of their learning outcomes (Theobald, 2021). Furthermore, self-regulated learners display metacognitive awareness, engaging in reflection on their learning processes, evaluation of their performance, and identification of areas for improvement (Andrade and Evans, 2012). Through self-reflection, self-evaluation, and self-assessment, they continually refine their learning strategies, optimizing their overall learning outcomes.

Blended teaching can make students autonomous in their language learning process by planning to learn, having more pace for selecting and sequencing the video- and audio-based content, possessing ownership, making decisions, enhancing higher-order learning skills, and observing learning to support self-regulated learning strategies (Lai and Hwang, 2016; Tan et al., 2017; Van Laer and Elen, 2017; Lee and Choi, 2019; Shih and Huang, 2020; Fathi et al., 2023). Moreover, an online flipped classroom, as a blended learning strategy, can offer authentic, meaningful, and personal materials, offer learners control and provide sufficient scaffolding and opportunities for interaction, reflection, and cooperation (Van Laer and Elen, 2017).

Within the context of online flipped classrooms, students are called upon to employ a spectrum of self-regulated learning strategies, encompassing cognitive, metacognitive, and behavioral dimensions, to effectively navigate their pre-class tasks and subsequently participate in in-class sessions (Geduld, 2016). The cognitive facet entails strategies such as rehearsing, organizing, transforming, and expanding knowledge, while metacognitive strategies involve goal setting and performance monitoring. Additionally, behavioral aspects encompass time and resource management as well as note-taking practices (Karlen, 2016). These multifaceted strategies converge to form a cohesive skill set crucial for successful EFL learning within the dynamic landscape of online flipped classrooms.

Our focus on the interaction between flipped classrooms and self-regulated learning not only acknowledges the evolving demands placed upon learners but also sheds light on the symbiotic relationship between instructional methodology and cognitive autonomy. While prior research has indeed identified predictive links between self-regulated strategies and flipped classrooms (Van Alten et al., 2020; Öztürk and Çakıroğlu, 2021), our study seeks to extend this understanding by validating and strengthening these insights within a distinct educational setting. To this end, this study aims to examine how online flipped classrooms can influence EFL learners’ reading comprehension and self-regulated learning strategies quantitatively and explore its role in students’ perception qualitatively.

### The present study

Although previous studies have explored the effectiveness of flipped instruction and online learning in improving language skills, there remains a need for more empirical research, particularly in the form of comparative studies that directly compare different instructional methods. This current study aims to address this research gap by investigating the effectiveness of three teaching methods–Online Flipped Instruction (OFI), Flipped Instruction (FI), and Traditional Instruction (TI)–in enhancing L2 reading comprehension performance and self-regulation among students.

Through the examination of online collaborative flipped instruction in the OFI group, traditional flipped instruction in the FI group, and conventional instruction in the TI group, this study will assess the impact of each method on students’ L2 reading comprehension performance and self-regulation. Via directly comparing these three approaches, the study will generate empirical evidence to identify the most effective teaching method for enhancing students’ language learning outcomes. Against this backdrop, this study aims to answer the following research questions:

1. What are the comparative effects of the OFI, FI, and TI methods on L2 reading comprehension performance?
2. What are the comparative effects of the OFI, FI, and TI methods on L2 reading self-regulation?
3. Are there significant differences in online learning behaviors and objective performances between students in the OFI and FI groups?

By answering these research questions, this study aims to contribute to the existing literature by providing empirical evidence on the comparative effectiveness of different instructional methods in promoting L2 reading comprehension and self-regulated learning of Chinese EFL learners. The rationale behind the emphasis on this specific student cohort (i.e., EFL learners) is rooted in the recognition that diverse factors, including cultural, linguistic, and educational backgrounds, can shape the implementation and outcomes of instructional methodologies. The intricacies of English language acquisition for Chinese learners (Wu, 2001), coupled with the demands of academic reading skills, render this population particularly intriguing for investigation. By delving into the experiences and responses of Chinese English learners within the realm of online collaborative flipped classrooms, this study aims to unearth insights that can inform tailored pedagogical strategies, curriculum design, and instructional support.

## Method

### Participants

The participants in this research were 71 EFL students, aged between 18 to 30 years old, who were enrolled in an English language course at a large language institute in mainland China. The majority of the participants were females (*n* = 45, 63.4%) and the rest of the students were males (*n* = 26, 36.6%). The participants had varying educational backgrounds, with most of them holding a high school diploma or equivalent (*n* = 54, 76.1%) and the remaining participants had a bachelor’s degree (*n* = 17, 23.9%). Participants’ proficiency level was determined based on the standardized placement test, Test of English for International Communication (TOEIC; Woodford, 1982), which is widely employed to evaluate the English proficiency of non-native speakers. Participants with an intermediate proficiency level (score range between 550 and 780) were included in the study. The participants were divided into three classes who were randomly assigned to a traditional instruction (TI) group (*n* = 24), and two experimental groups: Flipped Instruction (FI) group (*n* = 22) and Online Flipped Instruction (OFI) group (*n* = 25).

While addressing potential concerns regarding the sample size, insights were drawn from the recommendations of American Council on the Teaching of Foreign Languages (2010), which suggest that an optimal class size of approximately 15 students is advisable for facilitating collaborative learning activities effectively, especially within student-centered educational contexts. However, it’s worth noting that collaborative learning can still be implemented with larger class sizes, such as the 22 students in our study. To accommodate the larger class size while adhering to the principles of collaborative learning, we strategically designed the flipped instruction and online flipped instruction approaches, ensuring that they were conducive to group interactions and active engagement. This approach aimed to maintain the quality and effectiveness of collaborative learning, even with a larger number of students, thereby enhancing the reliability and validity of the research findings.

## Measures

### Reading comprehension

The participants’ reading comprehension ability was measured using the IELTS reading test (University of Cambridge ESOL Examinations, 2011). The IELTS reading test consists of three sections, each containing one long reading passage with increasing difficulty, followed by a set of multiple-choice questions. The IELTS Academic Reading test is a standardized assessment that consists of 40 questions and is administered within a strict time limit of 60 min. The test aims to assess the participants’ ability to comprehend and analyze academic English texts. The IELTS reading test has been extensively employed in L2 research, and has demonstrated good reliability and validity in measuring reading comprehension ability.

According to Weir and O’Sullivan (2017), the post-1989 evolution of IELTS primarily involves the transformation of the initial ELTS into a legitimate, psychometrically sound, high-stakes assessment with the capacity for widespread global administration on a large scale. Moreover, current data from the IELTS website (IELTS, 2021) provides comprehensive statistics for the test forms administered in 2019. Specifically, for the Reading section, the reported reliability coefficient stands at a robust value of 0.92, within a confidence interval of 0.90 to 0.93.

### Self-regulated learning strategy use questionnaire

The Self-Regulated Learning (SRL) questionnaire utilized in this study was developed by Tse et al. (2022) and was based on Zimmerman’s (2000) cyclical phases model. The questionnaire comprised 13 statements that assessed the employment of SRL strategies in English reading. The statements were formulated according to three categories of SRL strategies: planning, monitoring, and evaluating. Planning involved activities such as previewing reading tasks and setting goals prior to reading, whereas monitoring referred to checking and monitoring one’s reading progress and performance. Evaluating concerned the assessment of learning outcomes and the effectiveness of strategies. Participants rated all items using a 4-point Likert scale ranging from 1 (never or almost never) to 4 (every day or almost every day). The SRL strategy use questionnaire exhibited robust internal consistency, attaining a commendable coefficient of reliability at *r* = 0.86 within the context of this study.

### Online quizzes

To thoroughly assess the participants’ engagement with the course materials and their understanding of the content, a series of online quizzes were administered on a weekly basis to both the OFI and FI groups. These quizzes were thoughtfully designed to serve multiple purposes: to aid participants in their preparation for the subsequent in-class activities, to reinforce their comprehension of the assigned video lessons, and to evaluate their grasp of the core concepts relevant to the course.

Each weekly quiz comprised a carefully curated set of questions, ranging from 15 to 25 in number. These questions encompassed a variety of formats, including multiple-choice and short-answer questions, and were meticulously aligned with the main topics covered in the video lessons. Importantly, the questions were intentionally tailored to bridge the gap between the video content and the overarching theme of English reading comprehension. By incorporating elements of reading analysis, interpretation, and application, these quizzes aimed to foster a deeper understanding of the materials presented in the videos and to promote critical thinking in the context of L2 reading.

Throughout the 12-week course duration, a total of 10 quizzes were administered. The quizzes were strategically spaced to correspond with the course’s progression and to ensure that participants had the opportunity to revisit and consolidate their knowledge on a regular basis. The mean score obtained by each participant across these quizzes was computed as an additional metric of achievement. This mean score served to provide insights into the participants’ consistent performance and their evolving comprehension of the course materials. It is noteworthy that the quiz scores contributed significantly to the participants’ final grades, reflecting their competence in grasping the course content. Specifically, the final grades were calculated based on a comprehensive evaluation framework, which included various components. These components encompassed the overall participation score (50%), comprising class attendance (20%), quiz scores (20%), and assignments (10%). Additionally, the final grades considered the midterm test score (20%) and the final test (post-test) score (30%). This multifaceted approach to assessment aimed to holistically gage the participants’ progress, engagement, and achievement throughout the course.

### Online learning behavior

Following Fischer and Yang (2022), the present study examined three distinct online learning behaviors, encompassing regular online log-on time, group video-watching time, and total online log-on times. Each of these dimensions warrants attention and consideration within the context of our research. Firstly, regular online log-on time signifies the temporal commitment learners invest in engaging with the weekly assigned video lessons online. It reflects the extent to which students actively participate in the preparatory phase of flipped classrooms, where they access and assimilate instructional content independently before class. This dimension directly intersects with the flipped classroom model, as it measures the conscientiousness with which students approach their pre-class learning activities. Secondly, group video-watching time stands as a critical component of online learning behavior, capturing the duration during which participants in the experimental groups jointly engage in watching video lessons within small online groups. This dimension encapsulates the collaborative aspect of the flipped classroom approach, emphasizing the value of peer interaction and shared learning experiences. It is an essential component that furthers our understanding of how students engage with instructional materials and with each other, highlighting the interpersonal dimension of online learning. Lastly, total online log-on times amalgamate individual regular online log-on times with the time spent in small group video-watching sessions. This cumulative measure offers a comprehensive perspective on students’ overall engagement with online learning materials and activities. It underscores the holistic nature of online learning behavior, recognizing that effective learning in the digital realm encompasses both independent and collaborative dimensions.

Importantly, these dimensions of online learning behavior closely align with the tenets of self-regulated learning. SRL involves learners taking charge of their learning processes, which includes planning, monitoring, and evaluating their learning activities. The temporal commitment demonstrated through regular online log-on times resonates with the planning phase of SRL, where learners proactively engage with course materials and set the stage for effective learning. Group video-watching time correlates with the monitoring phase, as it reflects learners’ active involvement in tracking their progress through collaborative engagement. Lastly, the cumulative measure of total online log-on times speaks to the evaluation phase, wherein learners assess their learning strategies and the effectiveness of their collaborative endeavors.

Group video-watching time, on the other hand, exclusively captured the amount of time that the experimental group participants spent watching the video lessons in small online groups. Lastly, total online log-on times represented the cumulative measure, incorporating the individual regular online log-on times and the small group video-watching times of the participants.

To examine participants’ online learning behaviors, weekly video lessons and accompanying quizzes were uploaded onto the LMS used in this study. Both groups (i.e., FI and OFI) participants were instructed to watch the assigned videos on the LMS and complete the quizzes before attending class. The LMS automatically tracked each student’s log-on time for video viewing on a weekly basis. These log-on times were then aggregated to calculate the overall regular online log-on time for each participant throughout the course. Additionally, the students’ online activities, including video-watching and collaborative quiz sessions, were recorded. The duration of each online group session was calculated, resulting in the group video-watching time. This analysis provided insights into the students’ collective time spent watching the assigned videos and collaborating within the virtual environment, contributing to a comprehensive understanding of their online learning behaviors. To explore participants’ online learning behaviors, weekly video lessons and accompanying quizzes were uploaded to the LMS employed in this study. Both the Flipped Instruction and Online OFI groups were directed to view the designated videos on the LMS and take the quizzes prior to coming to class. The LMS automatically recorded students’ log-on times for video viewing on a weekly basis, which were then combined to determine their overall online log-on time throughout the course.

Furthermore, students’ online engagement, encompassing video-watching and collaborative quiz sessions, was meticulously logged. The duration of each group session was calculated, resulting in the compilation of group video-watching time. This analysis furnished valuable insights into the collective time students invested in viewing the assigned videos and engaging in collaboration within the virtual learning environment. Consequently, it contributed to a comprehensive comprehension of their online learning behaviors.

### Procedure

All three groups in the study were instructed by the same teacher, who had professional training in teaching English, specifically in academic reading skills. The course materials utilized in the study were focused on academic English reading skills, with particular emphasis on the learners’ proficiency in the IELTS academic reading test. The course lasted for 12 weeks, during which time all three groups received 180 min of instruction per week, distributed over two 90-min sessions. Pre-tests and post-tests were administered during the first and last week of the course to measure the progress of the learners.

In the traditional instruction (TI) group, the instructor employed the traditional ‘sage on the stage’ method to deliver the course materials and facilitate group exercises and activities. The classroom sessions primarily involved teacher-led instruction, group exercises, and activities. Pre- and post-test assessments were conducted by the teacher. In the flipped instruction (FI) and online flipped instruction (OFI) groups, the teacher played a more dynamic role, both inside and outside of the classroom. In the classroom, the instructor designed the curriculum, coordinated and supervised group activities, provided feedback and answered questions, as well as conducted pre- and post-test assessments. Beyond the confines of the traditional classroom setting, the educator meticulously curated and presented pre-designed video lessons, which were thoughtfully crafted to integrate reading comprehension exercises, textual analysis, and targeted reading strategies directly into the video content. Additionally, the teacher assumed responsibility for conceptualizing, producing, recording, and refining these tailored video lessons to ensure a comprehensive and effective approach to teaching reading skills and fostering self-regulated learning. Furthermore, the instructor efficiently oversaw the organization and administration of course materials within the educational institution’s designated learning management system. To facilitate online interactions, the teacher harnessed the capabilities of the Zoom Webinar video meeting web application. Moreover, during the OFI group sessions conducted virtually, the instructor delivered periodic feedback to foster an optimal learning environment.

In the weekly in-class sessions, the instructor began by providing brief announcements and instructions, as well as answering students’ questions. Then, for the remaining class time, the instructor divided students into groups and engaged them in various exercises and tasks to practice the weekly lesson content, which focused on reading skills and strategies. All learners were exposed to the identical in-class instruction. As part of their extracurricular learning, the FI students engaged in independent viewing of the designated course video lessons and subsequently undertook brief quizzes aligned with each instructional video.

In contrast to the other groups, the OFI students were purposefully grouped into small cohorts, consisting of no more than four individuals. These smaller groups fostered a conducive environment for collaborative learning as they collectively watched the assigned weekly video lesson online. Throughout these sessions, the OFI students actively engaged in collaborative discussions, exchanging ideas, and jointly completing the video quizzes, thereby reinforcing their understanding of the material as a unified entity. To facilitate seamless communication and interaction during their online video sessions, the small OFI groups effectively utilized Google Hangouts, leveraging its features for synchronous video conferencing and real-time collaboration. Consequently, their sessions were recorded and securely submitted to the instructor for meticulous review and insightful feedback.

It is noteworthy that the OFI students were explicitly informed of the significance of their weekly online video sessions. They were apprised that these sessions would be thoughtfully reviewed and deliberated upon during the subsequent in-person sessions held on a weekly basis. This ensured that the insights, discussions, and collaborative efforts from the online environment seamlessly integrated with the face-to-face instructional setting, fostering continuity and cohesion in the students’ learning experiences. In contrast, the TI students followed a different approach during their weekly lessons. These sessions encompassed a combination of in-class instruction focused on delivering the course content and a variety of engaging activities designed to enhance student participation and comprehension. Commencing each session, the instructor dedicated the initial half to comprehensive coverage of the lesson materials, providing necessary explanations and clarifications to support student learning. Subsequently, the latter portion of the session was dedicated to a range of interactive group activities and tasks, specifically designed to align with the lesson topics and skills covered in the assigned readings. This format emulated the structure and format adopted in the weekly in-class sessions of the FI and OFI groups, ensuring consistency and alignment across the instructional approaches employed throughout the study (Table 1).

**Table 1:** Main features of the reading intervention across experimental groups.

Instructional features	Traditional Instruction (TI)	Flipped Instruction (FI)	Online Flipped Instruction (OFI)
Teacher’s Role	‘Sage on the stage’ method	Dynamic role	Dynamic role
Curriculum design	Yes	Yes	Yes
Group activities	Yes	Yes	Yes
Feedback and Q&A	Yes	Yes	Yes
Pre- and post-tests	Yes	Yes	Yes
Video lessons	No	Yes	Yes
Online quizzes	No	Yes	Yes
Specialized video lessons	No	Some	Yes
Learning management system	No	No	Yes
Zoom webinar	No	No	Yes
Collaborative online sessions	No	No	Yes
Google hangouts integration	No	No	Yes
Recorded sessions review	No	No	Yes

Prior to the commencement of the study, all participants, regardless of their assigned experimental group, underwent pre-testing to establish baseline measurements of their reading comprehension abilities and SRL strategies. The pre-tests were conducted during the initial week of the course. Subsequently, upon completion of the 12-week course, post-tests were administered during the final week to assess the participants’ progress. The SRL strategy use questionnaire was administered at the beginning and end of the course to gage any changes in self-regulated learning behaviors and strategies. These assessments were conducted in a controlled classroom environment to ensure consistent testing conditions for all participants. Participants were instructed to respond to the pre- and post-tests and the SRL surveys with their utmost attention and sincerity, as their responses played a crucial role in evaluating the effectiveness of the instructional approaches employed in this study. The test and survey data were collected and analyzed to provide valuable insights into the impact of the different instructional methods on reading comprehension and self-regulated learning outcomes.

### Data analysis

To analyze the data collected for this study, the researchers used statistical software SPSS. The first research question was explored using a paired-sample t-test to compare the mean scores of the pre- and post-tests for the three groups (OFI, FI, and TI). Subsequently, to examine potential distinctions in the post-test scores among the groups, ANOVA was employed, supplemented by Fisher’s LSD post-hoc analysis. Regarding the third research question, an independent t-test was conducted to explore possible disparities in the online learning behaviors and objective performances, specifically the average online quiz scores and final course grades, between the two experimental flipped groups (OFI and FI).

### Results

The first research question aimed to investigate which teaching method–Traditional Instruction (TI), Flipped Instruction (FI), or Online Flipped Instruction (OFI)–yielded the most significant results in terms of the students’ L2 reading comprehension performance. To address this question, a paired-sample t-test was conducted to compare the pre-test and post-test scores of the students in each group. As seen in Table 2, the results revealed that all three groups showed significant improvement in their reading comprehension performance from pre-test to post-test. The mean scores of the students in the FI and OFI groups increased from pre-test to post-test, while the mean score of the TI group slightly decreased.

**Table 2:** Results of the paired-sample *t*-test for reading comprehension

	Pre-test		Post-test		*t*	*p*
	*M*	SD	*M*	SD		
TI	5.12	0.66	5.66	0.56	3.79**	0.005
FI	5.18	0.72	6.08	0.72	5.09***	0.000
OFI	4.99	0.83	6.41	0.69	6.74***	0.000

**p < 0.01, ***p < 0.001. TI, Traditional instruction; FI, Flipped Instruction; OFI, Online flipped instruction.

The paired-sample t-test results showed that all three groups showed significant improvement in their reading comprehension performance from pre-test to post-test (TI: *t* = 3.79, *p* = 0.005; FI: *t* = 5.09, *p* = 0.000; OFI: *t* = 6.74, *p* = 0.000). The OFI group showed the highest improvement, followed by the FI group, and then the TI group.

To further examine which teaching method had the most significant effect on the students’ reading comprehension performance, a one-way ANOVA was conducted. Table 3 presents the results of the ANOVA for reading comprehension. The results indicated a significant difference between the groups in terms of their reading comprehension performance [*F*(2, 68) = 3.61, *p* = 0.034].

**Table 3:** Results of ANOVA for reading comprehension.

	SS	df	MS	F	P
Between groups	3.02	2	1.51	3.61*	0.034
Within groups	21.32	68	0.43		
Total	24.35	70			

*p < 0.05.

*Post hoc* tests using the LSD method were conducted to determine which groups were significantly different from each other. Table 4 presents the results of the *post hoc* tests. The results showed that there was a significant difference in reading comprehension performance between the OFI and TI groups (*p* = 0.008), indicating that the OFI group had a significantly higher mean score than the TI group. Additionally, the results revealed a significant difference between the FI and OFI groups (*p* = 0.031), indicating that the OFI group had a slightly higher mean score than the FI group, although this difference was not as big as the difference between the OFI and TI groups. Finally, there was also a significant difference between the TI and FI groups (*p* = 0.023).

**Table 4:** Results of *post hoc* LSD.

					95% CI	
		Mean difference	SE	*p*	Lower	Upper
(I)	(J)	(I–J)				
TI	FI	0.42	0.191	0.023	0.042	0.798
OFI	TI	0.75	0.189	0.008	0.378	1.122
OFI	FI	0.33	0.194	0.031	−0.048	0.708

The second research question examined the differences in self-regulated learning strategies among students in the three teaching methods. Based on the results of the paired-sample t-test for self-regulated learning (Table 5), the two flipped teaching methods showed an increase in self-regulated learning from pre-test to post-test. However, the largest increase was observed in the OFI group (*M* = 3.15, SD = 0.68 to *M* = 4.35, SD = 0.67) with a significant t-value of 7.32 (*p* < 0.001). The FI group also showed a significant increase in self-regulated learning (*M* = 3.21, SD = 0.89 to *M* = 3.93, SD = 0.83) with a t-value of 4.30 (*p* < 0.01). The TI group, on the other hand, showed a non-significant increase in self-regulated learning (*M* = 3.28, SD = 0.71 to *M* = 3.41, SD = 0.75) with a t-value of 1.58 (*p* = 0.123).

**Table 5:** Results of the paired-sample t-test self-regulated learning.

	Pre-test		Post-test		*t*	*p*
	*M*	SD	*M*	SD		
TI	3.28	0.71	3.41	0.75	1.58	0.123
FI	3.21	0.89	3.93	0.83	4.30**	0.006
OFI	3.15	0.68	4.35	0.67	7.32***	0.000

**p < 0.01, ***p < 0.001.

Also, the results of the ANOVA for self-regulated learning in L2 reading (Table 6) indicate that there was a significant difference between the teaching methods in terms of their effect on self-regulated learning (*F* = 5.67, *p* = 0.012). The *post hoc* LSD analysis (Table 7) revealed that the OFI group had a significantly higher mean self-regulated learning score (*M* = 4.35, SE = 0.19) than both the FI group (*M* = 3.93, SE = 0.19) and the TI group (*M* = 3.41, SE = 0.19) with mean differences of 0.42 (*p* = 0.035) and 0.94 (*p* < 0.001), respectively. Additionally, the FI group had a significantly higher mean self-regulated learning score than the TI group with a mean difference of 0.52 (*p* = 0.015). Overall, it was found that both the FI and OFI teaching methods resulted in a greater increase in self-regulated learning in L2 reading compared to traditional instruction. Furthermore, the OFI method yielded the highest increase in self-regulated learning, suggesting that online flipped classrooms can be a beneficial approach for enhancing students’ self-regulation in L2 reading.

**Table 6:** Results of ANOVA self-regulated learning in L2 reading.

	SS	df	MS	F	*P*
Between groups	4.22	2	2.11	5.67*	0.012
Within groups	36.75	68	0.64		
Total	40.97	70			

*p < 0.05.

**Table 7:** Results of *post hoc* LSD.

					95% CI	
		Mean difference	SE	*p*	Lower	Upper
(I)	(J)	(I–J)				
TI	FI	0.52	0.192	0.015	0.097	0.943
OFI	TI	0.94	0.191	0.000	0.516	1.364
OFI	FI	0.42	0.196	0.035	0.026	0.814

The third research question addressed in this study was whether there were significant differences in the online learning behaviors and objective performances of OFI and FI students. Based on the results of the independent-samples *t*-tests (see Table 8) provided, there were significant differences in the total online log-on time, online quiz score, and final score between the FI and OFI students. The OFI students had a significantly higher total online log-on time (*M* = 9.23 h, SD = 3.27) than the FI students (*M* = 2.96 h, SD = 0.79), *t* = −5.234, *p* < 0.001. The OFI students also had a significantly higher online quiz score (*M* = 89, SD = 16.54) than the FI students (*M* = 64, SD = 14.23), *t* = −4.751, p < 0.001, and a significantly higher final score (*M* = 88, SD = 14.97) compared to the FI students (*M* = 69, SD = 11.29), *t* = −3.272, *p* = 0.008. These results suggest that the OFI students had better objective performances than the FI students in terms of online quiz and final score, and also spent more time online overall.

**Table 8:** Results of the independent-samples *t*-test.

	Group	*M*	SD	*t*	*p*
Total online log-on time (hours)	FI	2.96	0.79	– 5.234***	0.000
	OFI	9.23	3.27		
Online quiz score	FI	64	14.23	– 4.751***	0.000
	OFI	89	16.54		
Final score	FI	69	11.29	– 3.272**	0.008
	OFI	88	14.97		

**p < 0.01, ***p < 0.001.

## Discussions

The arrival of student-centered language education shed some light on the significance of implementing blended learning in EFL classes. One such developmental move was an online flipped classroom that could foster collaboration and cooperation, scaffolding (Topping and Ehly, 1998) and problem-solving (Barrows, 1996), and flexibility (Michael, 2006). Thus, the present study aimed to compare the effectiveness of traditional instruction (TI), flipped instruction (FI), and online flipped instruction (OFI) on the reading comprehension and self-regulated learning strategies of EFL learners. The findings of the study revealed that both FI and OFI instructional methods were more effective in improving students’ reading comprehension scores compared to traditional instruction. Furthermore, the OFI method showed significant improvements in students’ SRL strategy use compared to the other two groups. These results suggest that incorporating flipped and online flipped instruction into EFL reading instruction may enhance students’ learning outcomes.

One of the noteworthy findings of this study was the positive impact of online flipped classrooms on the reading comprehension abilities of EFL learners. The success of this approach can be attributed to a range of effective strategies employed. These encompass assigning videos and reading tasks for completion outside the traditional classroom, facilitating collaborative reading tasks through online communication platforms, and fostering reflection and self-evaluation of reading comprehension skills. These strategies have been supported by prior research, demonstrating increased motivation, engagement, and productivity within the classroom (Strelan et al., 2020; Vitta and Al-Hoorie, 2020).

These results can be attributed to a combination of well-founded strategies that EFL learners can effectively employ to enhance their reading performance in the context of flipped classrooms. Firstly, the practice of assigning videos and reading tasks for completion outside of the traditional classroom plays a pivotal role. This approach encourages learners to take ownership of their learning process, fostering independence and self-directed study habits. By engaging with course materials independently, students have the opportunity to delve deeper into the content, preparing them for more meaningful in-class discussions and activities. Secondly, the utilization of collaborative reading tasks facilitated through online communication platforms enhances the learning experience. It promotes peer interaction and shared exploration of texts, enabling learners to benefit from diverse perspectives and insights. This collaborative dimension not only enriches their understanding of the reading materials but also cultivates crucial communication skills in an online context, aligning with the demands of the digital age. Furthermore, the practice of facilitating peer feedback exchange during reading activities fosters a constructive learning environment. Learners actively contribute to each other’s growth by providing valuable insights and critiques. This not only bolsters their comprehension but also encourages a culture of continuous improvement and mutual support. In addition, individual and collective reflection on reading practices is integral to the success of flipped classrooms. Encouraging learners to assess their own reading comprehension skills and engage in group discussions about their strategies encourages metacognitive awareness. It empowers them to adapt and refine their approaches to reading, leading to more effective comprehension. Moreover, the promotion of self-evaluation of reading comprehension skills empowers learners to take charge of their progress. By regularly assessing their own understanding and identifying areas for improvement, students become more self-aware and accountable for their learning outcomes.

Finally, providing opportunities for communicative activities beyond the confines of the classroom solidifies the positive outcomes of online flipped classrooms. These activities allow students to apply their reading comprehension skills in real-world contexts, reinforcing their practical utility. As a result, they become more motivated, engaged, reflective, and productive within the classroom environment. These findings align with the research of Strelan et al. (2020) and Vitta and Al-Hoorie (2020), underscoring the effectiveness of these instructional approaches. By implementing these strategies, educators can harness the power of online flipped classrooms to elevate student motivation, engagement, reflection, and productivity. Ultimately, learners actively participate in critical discussions, engage in the negotiation of meaning, and emerge with a deeper understanding of the reading materials.

The effectiveness of these strategies is substantiated by prior research (Guo, 2019; Fulgueras and Bautista, 2020; Mohammad Hosseini et al., 2020; Shih and Huang, 2020; Samiei and Ebadi, 2021; Yulian, 2021; Fischer and Yang, 2022) which has consistently demonstrated their efficacy in improving EFL learners’ skills in general and L2 reading in particular. Also, the results of the study are consistent with previous research that has found that flipped instruction can lead to increased student engagement and achievement (Strayer, 2012; Bergmann and Sams, 2014; Stöhr et al., 2020; Turan and Akdag-Cimen, 2020). Flipped instruction allows students to take control of their learning by providing them with access to course materials outside of class and enabling them to review and study at their own pace. The results of the current study also support the findings of previous research that has found that online learning can be an effective method of instruction (Bernard et al., 2009).

It was also revealed that online flipped instruction had a more significant effect on L2 reading comprehension of the EFL participants than flipped instruction. This difference can be attributed to several key factors. Firstly, the essence of the flipped classroom model, as conceptualized by Bergmann and Sams (2014) and Su Ping et al. (2020), revolves around providing students with pre-class access to instructional materials. This empowers them to progress through the content at their own pace, ensuring a foundational understanding before engaging in more dynamic and participatory in-class activities. This advantage is further magnified when executed through online platforms (Stöhr et al., 2020), where students can efficiently allocate their in-class time for focused practice and interactive learning experiences (Hew et al., 2020).

Additionally, a body of prior research underscores the potency of technology-based instruction, particularly the use of online materials and multimedia resources, in augmenting language learning outcomes (González-Lloret, 2019; Jain et al., 2023). The dynamic and interactive nature of technology-supported learning environments can substantially contribute to language skill development (Yang et al., 2021). By providing our students with accessible online materials tailored to their individual learning pace, the online flipped instruction approach likely fostered a deeper engagement with the content and a more effective honing of their reading skills.

Furthermore, the integration of multimedia resources within our online materials played a pivotal role in sustaining student engagement and motivation (Shin et al., 2020). This multimedia-rich environment not only catered to diverse learning preferences but also injected an element of interactivity, capturing and sustaining student interest throughout the learning process.

Previous research also supported that reading comprehension could be enhanced by engaging learners in an online flipped classroom model (Karimi and Hamzavi, 2017; Hashemifardnia et al., 2018; Samiei and Ebadi, 2021). As reading comprehension involves associating the background knowledge with the reading text, EFL learners who are connected to the internet and can search around any topic become able to create the background knowledge before starting their reading due to their time flexibility outside of class. Participating in online flipped classrooms empowers EFL learners, providing them with the agency and autonomy to make informed decisions and take purposeful actions in their reading practices (Fulgueras and Bautista, 2020). This pedagogical approach fosters a sense of ownership and responsibility in learners, motivating them to adapt and refine their reading strategies, and explore novel approaches and techniques (Fischer and Yang, 2022). Furthermore, online flipped classrooms cultivate a positive attitude toward the inherent challenges of comprehending texts, encouraging learners to perceive these challenges as opportunities for personal growth and deeper understanding (Samiei and Ebadi, 2021).

Moreover, EFL learners derive significant benefits from the opportunity to enhance their vocabulary and grammatical knowledge through engaging with various activities before, during, and after reading texts (Turan and Akdag-Cimen, 2020). They actively employ a range of strategies to strengthen their language proficiency, including the use of dictionaries to clarify unfamiliar words, the application of contextual cues to predict and infer meaning, engaging in peer discussions to seek clarification and deepen understanding, and employing effective organizational strategies such as rehearsal, rereading, and summarization. By skillfully utilizing these cognitive and metacognitive strategies, learners cultivate an enriched reading process that facilitates comprehensive comprehension and fosters critical thinking abilities (Karimi and Hamzavi, 2017; Fulgueras and Bautista, 2020; Samiei and Ebadi, 2021; Yulian, 2021; Li et al., 2022; Liu et al., 2022). Furthermore, EFL learners can reach opportunities to discuss and negotiate the reading points, ideas, difficulties, and strategies and engage in problem-solving and information-exchange communicative activities while they are in the classes (Kim et al., 2017; Zarrinabadi and Ebrahimi, 2018). Additionally, Gok et al. (2021) discovered that EFL learners’ classroom anxiety and foreign language reading anxiety could be deceased during the flipped classroom, and they could be more motivated to comprehend reading text (Jiang et al., 2021). In the same vein, Hashemifardnia et al. (2018) studied how flipped classrooms predicted junior high school students’ reading comprehension. They identified that online flipped classrooms could considerably affect reading comprehension.

Another finding of this study was the significance of online flipped classrooms on self-regulated learning strategies of learners since learners can gradually learn how to acquire, organize, reflect, and appraise their own learning practices (Lai and Hwang, 2016). EFL learners need to plan their tasks and exercises outside of class, set aims and objectives and develop their understanding of their styles of learning (metacognitive strategy), recognize and boost their efforts, consolidate content and input (behavioral and motivational), observe and evaluate their learning (self-evaluation) (Paris and Paris, 2001; Artino and Stephens, 2009; Zimmerman and Moylan, 2009; Kistner et al., 2010; Kramarski et al., 2013; Panadero and Alonso-Tapia, 2014). Also, as learners need to be actively involved in flipped classrooms to make decisions, become autonomous, take ownership, know their competence, regulate their learning process, perceive the intended meanings, and link the personal and environmental factors, they can reach more incredible self-regulated learning strategies (Zimmerman, 2000; Pintrich, 2004; Pajares, 2009). Thus, online flipped classrooms can benefit EFL learners’ reading comprehension and self-regulated strategies. More specifically, online collaborative learning can enhance SRL strategies by providing students with opportunities to work together, reflect on their learning, and engage in metacognitive processes (Zimmerman, 2000; Dabbagh and Kitsantas, 2012; Kim et al., 2017; Lei et al., 2022). online instruction provides learners with greater autonomy and control over their learning, allowing them to engage in self-regulated learning activities such as setting goals, monitoring progress, and adapting strategies as needed (Dabbagh and Kitsantas, 2012). In contrast, traditional classroom-based instruction can limit learner autonomy and control, as learners may be subject to the pace and structure of the lesson set by the teacher.

Another finding of this study was the significance of online flipped classrooms on self-regulated learning strategies of EFL learners. This finding can be attributed to several key factors, each underpinned by sound pedagogical principles. To begin with, the framework of online flipped classrooms inherently promotes the gradual development of SRL skills among learners (Lai and Hwang, 2016). This gradual evolution is facilitated as learners are encouraged to acquire, organize, reflect upon, and appraise their own learning practices over time. In the context of EFL learning, this process necessitates students to meticulously plan their tasks and exercises outside the traditional class setting, establish clear aims and objectives, and cultivate a nuanced understanding of their unique learning styles–a core facet of metacognitive strategy (Paris and Paris, 2001; Artino and Stephens, 2009; Zimmerman and Moylan, 2009; Kistner et al., 2010; Kramarski et al., 2013; Panadero and Alonso-Tapia, 2014).

Moreover, active participation in online flipped classrooms empowers learners to make informed decisions, fostering autonomy, ownership of their learning journey, and an acute awareness of their competence–all central tenets of SRL (Zimmerman, 2000; Pintrich, 2004; Pajares, 2009). Within this dynamic educational context, students are encouraged to regulate their learning processes, decipher intended meanings, and establish connections between personal and environmental factors, thereby further enriching their repertoire of SRL strategies. The advantages of online collaborative learning, a key component of our online flipped classrooms, further bolster the enhancement of SRL strategies. Collaborative learning environments provide students with invaluable opportunities to work together, engage in reflective practices, and participate in metacognitive processes (Zimmerman, 2000; Dabbagh and Kitsantas, 2012; Kim et al., 2017; Lei et al., 2022). Through collaborative endeavors, students gain insights from their peers, engage in discussions that require them to reflect on their learning, and employ metacognitive strategies to evaluate their comprehension and learning progress.

Additionally, online instruction, in general, affords learners greater autonomy and control over their learning experiences. This increased agency empowers students to engage in various SRL activities, including setting meaningful goals, monitoring their progress, and adapting strategies as needed (Dabbagh and Kitsantas, 2012). In contrast, traditional classroom-based instruction may inadvertently curtail learner autonomy and control, as students often find themselves subject to the predetermined pace and structure of lessons set by the teacher.

Additionally, the flipped instruction approach can provide learners with opportunities to engage in self-regulated learning activities such as previewing, reviewing, and reflecting on materials before and after class (Bergmann and Sams, 2014). However, in a traditional flipped instruction approach, learners may be limited to engaging in these activities outside the classroom, which may not be conducive to developing self-regulated learning skills. In contrast, online flipped instruction can provide learners with ongoing access to materials, resources, and feedback, allowing them to engage in self-regulated learning activities both inside and outside the classroom (Galway et al., 2014; Jia et al., 2023).

Finally, the findings revealed that the OFI students demonstrated superior online learning behaviors and objective performances than the FI students. One key difference was the amount of time the students spent engaging with the online course materials. These differences can be attributed to several key factors, each contributing to the enhanced performance of the OFI students. First and foremost, the notable disparity in the amount of time dedicated to engaging with online course materials between the OFI and FI groups is a pivotal finding. OFI students invested significantly more time in their learning endeavors compared to the FI students. This disparity suggests that the inclusion of the online component within the OFI group acted as a motivating factor, encouraging students to spend more time interacting with course materials. The online collaborative flipped classroom approach inherently promotes active learning, fostering a sense of responsibility and autonomy among students (Burke and Fedorek, 2017; Tang et al., 2023). This encourages learners to delve deeper into the subject matter and allocate more time to their studies.

Moreover, the higher scores achieved by the OFI students on online quizzes provide additional insights. This outcome underscores the effectiveness of the online collaborative flipped classroom approach in the OFI group, as it enabled students to grasp the subject matter more profoundly. The combination of pre-learning through online resources and the collaborative online component in the OFI group likely facilitated a more comprehensive understanding of the material (Ng et al., 2022). This approach allowed students to review content at their own pace, seek clarifications as needed, and actively engage in collaborative activities with their peers–factors that collectively contributed to improved quiz performance. Furthermore, the superior overall final scores attained by the OFI students compared to the FI students signify not only their proficiency in online quizzes but also their enhanced performance on the final assessment. This result suggests that the benefits observed in the online component of the OFI approach transcended the realm of quizzes and extended to broader assessments. The amalgamation of flipped instruction and the collaborative online component likely played a synergistic role in fostering improved learning outcomes.

## Conclusion and implications

This study investigated the effectiveness of an online collaborative flipped classroom approach in improving English reading skills and self-regulated learning among Chinese EFL learners. The results demonstrated that both the FI and OFI groups outperformed the traditional group in terms of reading comprehension and self-regulated learning. Moreover, the OFI students exhibited superior online learning behaviors and objective performances compared to the FI students, spending more time engaging with the course materials and achieving higher scores on online quizzes and final assessments. These findings highlight the potential benefits of integrating flipped instruction and online collaboration in L2 instruction.

The findings of this study have several implications for educators and researchers in the field of language instruction. Firstly, the incorporation of an online collaborative flipped classroom approach has the potential to enhance students’ engagement, learning outcomes, and overall performance in English reading skills. Educators are encouraged to consider adopting similar instructional methods that promote active learning, self-regulated strategies, and collaborative learning opportunities. Secondly, the study highlights the effectiveness of combining online resources with flipped instruction to create a blended learning environment. Integrating online components allows learners to access course materials flexibly, review content at their own pace, and engage in collaborative activities, thereby enhancing their learning experience.

Moreover, the online collaborative flipped classroom model promoted self-regulated learning behaviors, such as time management, goal-setting, and progress monitoring. Educators should provide explicit instruction and support in developing self-regulated learning skills to empower learners to take ownership of their learning process. Furthermore, the study underscores the importance of leveraging technology for language instruction. Online platforms, interactive tools, and multimedia resources facilitate learner engagement, provide opportunities for authentic practice, and enable effective monitoring of students’ progress. In terms of future research, it would be valuable to explore the long-term effects of the online collaborative flipped classroom approach on language acquisition and the transferability of skills. Additionally, investigating the impact of this instructional model on other language skills and diverse learner populations would contribute to a more comprehensive understanding of its potential benefits.

Finally, as the educational landscape undergoes profound transformations driven by technological advancements, it is paramount to recognize the sweeping changes shaping modern education. These shifts span diverse domains, encompassing innovative applications of artificial intelligence technology, the adoption of tailored models for digital transformation, and the exploration of how online platforms impact learner satisfaction (García-Morales et al., 2021; Zarifis and Efthymiou, 2022; Jain et al., 2023; Widayanti and Meria, 2023). Embracing this broader perspective, our research findings naturally align within the dynamic realm of this educational transformation, offering insights that resonate with the evolving fabric of contemporary learning.

Despite the valuable insights garnered from this study, it is essential to acknowledge several limitations. Firstly, the sample size utilized in this study was relatively small, comprising participants from a specific educational context in China. It is important to acknowledge that the relatively modest participant count of 71 EFL students may be subject to constraints in statistical power, particularly for detecting smaller effect sizes. While the sample size was chosen to achieve a balance between practical feasibility and the desire for meaningful statistical analyzes, future studies with larger cohorts could provide a more robust foundation for further validating the outcomes observed in this research. Secondly, the study’s duration was limited to a 12-week period, potentially constraining the ability to observe any long-term effects of the instructional intervention. Future research endeavors should consider implementing the intervention over an extended timeframe to thoroughly assess the sustainability of the observed benefits.

Moreover, it is imperative to recognize that this study relied solely on quantitative data and employed self-report measures to evaluate self-regulated learning strategies and online learning behaviors. While self-report measures are commonly employed, it is crucial to acknowledge their inherent subjectivity and susceptibility to response biases. Augmenting the self-report data with objective measures, such as direct observation or behavioral tracking, would enhance the robustness and validity of the findings, enabling a more comprehensive understanding of learners’ engagement and behaviors. Furthermore, it is noteworthy that the findings of this study may have been influenced by the specific context in which it was conducted, encompassing factors such as cultural background, educational system, and institutional support. These contextual elements have the potential to impact the efficacy of the employed instructional approaches. Consequently, caution should be exercised when attempting to generalize the findings to diverse contexts. Future research endeavors should strive to investigate the effectiveness of online collaborative flipped instruction in a range of educational settings, thereby enabling a more nuanced understanding of its applicability.

## Data availability statement

The raw data supporting the conclusions of this article will be made available by the authors, without undue reservation. Requests to access these datasets should be directed to YW, wangying0635@163.com.

## Ethics statement

The studies involving humans were approved by Department of Foreign Language, Liaocheng University Dongchang College, Liaocheng 252000, Shandong, China. The studies were conducted in accordance with the local legislation and institutional requirements. The participants provided their written informed consent to participate in this study.

## Author contributions

YW: Conceptualization, Data curation, Investigation, Methodology, Project administration, Resources, Visualization, Writing – original draft, Writing – review & editing.

## References

[ref2] American Council on the Teaching of Foreign Languages. (2010). *Maximum class size*. Available at: https://www.actfl.org/search/node/class%20size. (Accessed May 2, 2016).

[ref1] Amer M. (2014). A study of learners’ usage of a mobile learning application for learning idioms and collocations. CALICO J. 31, 285–302. doi: 10.11139/cj.31.3.285302

[ref3] Andrade M. S. Evans N. W. (2012). Principles and practices for response in second language writing: developing self-regulated learners. Abingdon: Routledge.

[ref4] Artino A. R. Stephens J. M. (2009). Academic motivation and self-regulation: a comparative analysis of undergraduate and graduate students learning online. Internet High. Educ. 12, 146–151. doi: 10.1016/j.iheduc.2009.02.001

[ref5] Barrows H. S. (1996). Problem-based learning in medicine and beyond: a brief overview. New Dir. Teach. Learn. 1996, 3–12. doi: 10.1002/tl.37219966804

[ref6] Basilaia G. Kvavadze D. (2020). Transition to online education in schools during a SARS-CoV-2 coronavirus (COVID-19) pandemic in Georgia. Pedagog. Res. 5, 1–9. doi: 10.29333/pr/7937

[ref7] Bergmann J. Sams A. (2014). Flipped learning: Gateway to student engagement. Washington, DC: International Society for Technology in Education.

[ref9002] Bernard R. M. Abrami P. C. Borokhovski E. Wade C. A. Tamim R. M. Surkes M. A.. (2009). A meta-analysis of three types of interaction treatments in distance education. Rev. Educ. Res. 79, 1243–1289. doi: 10.3102/0034654309333844

[ref9] Burke A. S. Fedorek B. (2017). Does “flipping” promote engagement?: a comparison of a traditional, online, and flipped class. Act. Learn. High. Educ. 18, 11–24. doi: 10.1177/1469787417693487

[ref10] Butt A. (2014). Student views on the use of a flipped classroom approach: evidence from Australia. Bus. Educ. Accreditat. 6, 33–43.

[ref11] Çakıroğlu Ü. Öztürk M. (2017). Flipped classroom with problem based activities: exploring self-regulated learning in a programming language course. J. Educ. Technol. Soc. 20, 337–349.

[ref12] Chen Hsieh J. S. Wu W. C. V. Marek M. W. (2017). Using the flipped classroom to enhance EFL learning. Comput. Assist. Lang. Learn. 30, 1–21. doi: 10.1080/09588221.2015.1111910

[ref14] Critz C. M. Knight D. (2013). Using the flipped classroom in graduate nursing education. Nurse Educ. 38, 210–213. doi: 10.1097/NNE.0b013e3182a0e56a, PMID: 23969751

[ref15] Crouch C. H. Watkins J. Fagen A. P. Mazur E. (2007). Peer instruction: engaging students one-on-one, all at once. Res. Based Reform Univ. Phys. 1, 40–95.

[ref9001] Dabbagh N. Kitsantas A. (2012). Personal learning environments, social media, and self-regulated learning: A natural formula for connecting formal and informal learning. Internet. High. Educ. 15, 3–8. doi: 10.1016/j.iheduc.2011.06.002

[ref16] Davis C. (2013). “Flipped or inverted learning: strategies for course design” in Enhancing instruction with visual media: Utilizing video and lecture capture. eds. Smyth E. G. Volker J. X. (Hershey, PA: IGI Global), 241–265.

[ref17] Fathi J. Mohammaddokht F. Afzali M. (2023). Exploring Iranian EFL teachers’ attitudes toward the use of learning management systems in English classes. Íkala Rev. Lenguaje Cult. 28, 30–48. doi: 10.17533/udea.ikala.v28n1a02

[ref18] Fathi J. Naghshbandi Z. Mohamadi P. (2021). The effect of a flipped writing classroom on writing performance and self-regulation of Iranian EFL learners. Lang. Relat. Res. 12, 627–659. doi: 10.29252/LRR.12.4.20

[ref19] Fathi J. Rahimi M. (2022). Examining the impact of flipped classroom on writing complexity, accuracy, and fluency: a case of EFL students. Comput. Assist. Lang. Learn. 35, 1668–1706. doi: 10.1080/09588221.2020.1825097

[ref20] Fathi J. Rahimi M. Liu G. Z. (2023). A preliminary study on flipping an English as a foreign language collaborative writing course with video clips: its impact on writing skills and writing motivation. J. Comput. Assist. Learn. 39, 659–675. doi: 10.1111/jcal.12772

[ref21] Fischer I. D. Yang J. C. (2022). Flipping the flipped class: using online collaboration to enhance EFL students’ oral learning skills. Int. J. Educ. Technol. High. Educ. 19:15. doi: 10.1186/s41239-022-00320-2

[ref22] Fisher T. (2006). Educational transformation: is it, like ‘beauty’, in the eye of the beholder, or will we know it when we see it? Educ. Inf. Technol. 11, 293–303. doi: 10.1007/s10639-006-9009-1

[ref23] Fulgueras M. J. Bautista J. (2020). Flipped classroom: its effects on ESL learners’ critical thinking and Reading comprehension levels. Int. J. Lang. Lit. Stud. 2, 257–270. doi: 10.36892/ijlls.v2i3.228

[ref24] Galway L. P. Corbett K. K. Takaro T. K. Tairyan K. Frank E. (2014). A novel integration of online and flipped classroom instructional models in public health higher education. BMC Med. Educ. 14, 1–9. doi: 10.1186/1472-6920-14-181, PMID: 25169853 PMC4167261

[ref25] García-Morales V. J. Garrido-Moreno A. Martín-Rojas R. (2021). The transformation of higher education after the COVID disruption: emerging challenges in an online learning scenario. Front. Psychol. 12:616059. doi: 10.3389/fpsyg.2021.616059, PMID: 33643144 PMC7904694

[ref26] Geduld B. (2016). Exploring differences between self-regulated learning strategies of high and low achievers in open distance learning. Afr. Educ. Rev. 13, 164–181. doi: 10.1080/18146627.2016.1182739

[ref27] Gok D. Bozoglan H. Bozoglan B. (2021). Effects of online flipped classroom on foreign language classroom anxiety and reading anxiety. Comput. Assist. Lang. Learn. 36, 840–860. doi: 10.1080/09588221.2021.1950191

[ref28] González-Lloret M. (2019). Technology and L2 pragmatics learning. Annu. Rev. Appl. Linguist. 39, 113–127. doi: 10.1017/S0267190519000047

[ref29] Guo J. J. (2019). The use of an extended flipped classroom model in improving students’ learning in an undergraduate course. J. Comput. High. Educ. 31, 362–390. doi: 10.1007/s12528-019-09224-z

[ref30] Hasan M. K. Ibna Seraj P. M. Fakih A.-H. Klimova B. (2022). Conceptions and viewpoints of English as a foreign language undergraduate students towards flipped instructed classroom. Educ. Res. Int. 2022:6140246. doi: 10.1155/2022/6140246

[ref31] Hashemifardnia A. Namaziandost E. Shafiee S. (2018). The effect of implementing flipped classrooms on Iranian junior high school students' reading comprehension. Theory Pract. Lang. Stud. 8, 665–673. doi: 10.17507/tpls.0806.17

[ref32] Hew K. F. Jia C. Gonda D. E. Bai S. (2020). Transitioning to the “new normal” of learning in unpredictable times: pedagogical practices and learning performance in fully online flipped classrooms. Int. J. Educ. Technol. High. Educ. 17, 57–22. doi: 10.1186/s41239-020-00234-x, PMID: 34778516 PMC7750097

[ref33] Hung H. T. (2015). Flipping the classroom for English language learners to foster active learning. Comput. Assist. Lang. Learn. 28, 81–96. doi: 10.1080/09588221.2014.967701

[ref34] Hung H. T. (2017). Design-based research: redesign of an English language course using a flipped classroom approach. TESOL Q. 51, 180–192. doi: 10.1002/tesq.328

[ref35] IELTS. (2021). *Test performance*. Available at: https://www.ielts.org/en-us/for-researchers/test-statistics/test-performance.

[ref36] Jain A. Sharma P. Meher J. R. (2023). Effects of online platforms on learner's satisfaction: a serial mediation analysis with instructor presence and student engagement. Int. J. Inf. Learn. Technol. 2023:17. doi: 10.1108/IJILT-02-2023-0017

[ref37] Jeon E. H. Yamashita J. (2014). L2 reading comprehension and its correlates: a meta-analysis. Lang. Learn. 64, 160–212. doi: 10.1111/lang.12034

[ref38] Jia C. Hew K. F. Jiahui D. Liuyufeng L. (2023). Towards a fully online flipped classroom model to support student learning outcomes and engagement: a 2-year design-based study. Internet High. Educ. 56:100878. doi: 10.1016/j.iheduc.2022.100878

[ref40] Jiang L. Meng H. Zhou N. (2021). English learners’ readiness for online flipped learning: interrelationships with motivation and engagement, attitude, and support. Lang. Teach. Res. 2021:27459. doi: 10.1177/13621688211027459

[ref39] Jiang M. Y. C. Jong M. S. Y. Lau W. W. F. Chai C. S. Liu K. S. X. Park M. (2022). A scoping review on flipped classroom approach in language education: challenges, implications and an interaction model. Comput. Assist. Lang. Learn. 35, 1218–1249. doi: 10.1080/09588221.2020.1789171

[ref41] Karabulut-Ilgu A. Jaramillo Cherrez N. Jahren C. T. (2018). A systematic review of research on the flipped learning method in engineering education. Br. J. Educ. Technol. 49, 398–411. doi: 10.1111/bjet.12548

[ref42] Karimi M. Hamzavi R. (2017). The effect of flipped model of instruction on EFL learners’ reading comprehension: learners’ attitudes in focus. Adv. Lang. Literary Stud. 8, 95–103. doi: 10.7575/aiac.alls.v.8n.1p.95

[ref43] Karjanto N. Simon L. (2019). English-medium instruction Calculus in Confucian-heritage culture: flipping the class or overriding the culture? Stud. Educ. Eval. 63, 122–135. doi: 10.1016/j.stueduc.2019.07.002

[ref44] Karlen Y. (2016). Differences in students’ metacognitive strategy knowledge, motivation, and strategy use: a typology of self-regulated learners. J. Educ. Res. 109, 253–265. doi: 10.1080/00220671.2014.942895

[ref45] Kendeou P. Van den Broek P. Helder A. Karlsson J. (2014). A cognitive view of reading comprehension: implications for reading difficulties. Learn. Disabil. Res. Pract. 29, 10–16. doi: 10.1111/ldrp.12025

[ref46] Kiernan P. J. Aizawa K. (2004). Cell phones in task based learning are cell phones useful language learning tools? ReCALL 16, 71–84. doi: 10.1017/S0958344004000618

[ref47] Kim J. Park H. Jang M. Nam H. (2017). Exploring flipped classroom effects on second language learners’ cognitive processing. Foreign Lang. Ann. 50, 260–284. doi: 10.1111/flan.12260

[ref48] Kintsch W. (2012). “Psychological models of reading comprehension and their implications for assessment” in Measuring up: Advances in how we assess reading ability. eds. Sabatini J. P. Albro E. R. O’Reilly T. (Rowman and Littlefield Education: Plymouth)

[ref49] Kistner S. Rakoczy K. Otto B. Dignath-van Ewijk C. Büttner G. Klieme E. (2010). Promotion of self-regulated learning in classrooms: investigating frequency, quality, and consequences for student performance. Metacogn. Learn. 5, 157–171. doi: 10.1007/s11409-010-9055-3

[ref50] Kramarski B. Weiss I. Sharon S. (2013). Generic versus context-specific prompts for supporting self-regulation in mathematical problem solving among students with low or high prior knowledge. J. Cogn. Educ. Psychol. 12, 197–214. doi: 10.1891/1945-8959.12.2.197

[ref51] Lai C. L. Hwang G. J. (2016). A self-regulated flipped classroom approach to improving students’ learning performance in a mathematics course. Comput. Educ. 100, 126–140. doi: 10.1016/j.compedu.2016.05.006

[ref53] Lee G. Wallace A. (2018). Flipped learning in the English as a foreign language classroom: outcomes and perceptions. TESOL Q. 52, 62–84. doi: 10.1002/tesq.372

[ref52] Lee J. Choi H. (2019). Rethinking the flipped learning pre-class: its influence on the success of flipped learning and related factors. Br. J. Educ. Technol. 50, 934–945. doi: 10.1111/bjet.12618

[ref54] Lei X. Fathi J. Noorbakhsh S. Rahimi M. (2022). The impact of mobile-assisted language learning on English as a foreign language learners’ vocabulary learning attitudes and self-regulatory capacity. Front. Psychol. 13:872922. doi: 10.3389/fpsyg.2022.872922, PMID: 35800918 PMC9255592

[ref56] Lin C. H. Zhang Y. Zheng B. (2017). The roles of learning strategies and motivation in online language learning: a structural equation modeling analysis. Comput. Educ. 113, 75–85. doi: 10.1016/j.compedu.2017.05.014

[ref55] Li S. He J. Tao Y. Liu X. (2022). The effects of flipped classroom approach in EFL teaching: can we strategically use the flipped method to acquire communicative competence? Lang. Teach. Res. 2022:1575. doi: 10.1177/13621688221081575

[ref58] Liu C. Sands-Meyer S. Audran J. (2019). The effectiveness of the student response system (SRS) in English grammar learning in a flipped English as a foreign language (EFL) class. Interact. Learn. Environ. 27, 1178–1191. doi: 10.1080/10494820.2018.1528283

[ref57] Liu G. Z. Rahimi M. Fathi J. (2022). Flipping writing metacognitive strategies and writing skills in an English as a foreign language collaborative writing context: a mixed-methods study. J. Comput. Assist. Learn. 38, 1730–1751. doi: 10.1111/jcal.12707

[ref59] Maharsi I. Wijayanti Y. R. Astari T. R. (2021). Evaluating flipped classroom approach in EFL students reading classes. LLT J. 24, 92–102. doi: 10.24071/llt.v24i1.2768

[ref60] Mehring J. (2016). Present research on the flipped classroom and potential tools for the EFL classroom. Comput. Sch. 33, 1–10. doi: 10.1080/07380569.2016.1139912

[ref61] Meniado J. C. (2016). Metacognitive Reading strategies, motivation, and Reading comprehension performance of Saudi EFL students. Engl. Lang. Teach. 9, 117–129. doi: 10.5539/elt.v9n3p117

[ref62] Michael J. (2006). Where’s the evidence that active learning works? Adv. Physiol. Educ. 30, 159–167. doi: 10.1152/advan.00053.2006 17108243

[ref64] Mohammaddokht F. Fathi J. (2022). An investigation of flipping an English reading course: focus on reading gains and anxiety. Educ. Res. Int. 2022:62983. doi: 10.1155/2022/2262983

[ref63] Mohammad Hosseini H. Ejtehadi A. Mohammad Hosseini M. (2020). Flipping microlearning-based EFL classroom to enhance learners’ self-regulation. Lang. Teach. Res. Q. 20, 43–59. doi: 10.32038/ltrq.2020.20.03

[ref65] Murdock J. L. Williams A. M. (2011). Creating an online learning community: is it possible? Innov. High. Educ. 36, 305–315. doi: 10.1007/s10755-011-9188-6

[ref66] Ng D. T. Ng E. H. Chu S. K. (2022). Engaging students in creative music making with musical instrument application in an online flipped classroom. Educ. Inf. Technol. 27, 45–64. doi: 10.1007/s10639-021-10568-2, PMID: 34230800 PMC8249043

[ref67] Nilson L. B. (2023). Creating self-regulated learners: strategies to strengthen students’ self-awareness and learning skills. Abingdon: Taylor and Francis.

[ref68] Novak G. M. (2011). Just-in-time teaching. New Dir. Teach. Learn. 2011, 63–73. doi: 10.1002/tl.469

[ref69] Nursyahdiyah N. Dalimunte A. A. Daulay S. H. (2022). The implementation of flipped classroom in EFL reading during Covid-19 pandemic: Indonesian EFL students’ voices. Engl. Franca 6, 325–340. doi: 10.29240/ef.v6i2.5329

[ref70] O’Flaherty J. Phillips C. (2015). The use of flipped classrooms in higher education: a scoping review. Intern High. Educ. 25, 85–95. doi: 10.1016/j.iheduc.2015.02.002

[ref71] Öztürk M. Çakıroğlu Ü. (2021). Flipped learning design in EFL classrooms: implementing self-regulated learning strategies to develop language skills. Smart Learn. Environ. 8:2. doi: 10.1186/s40561-021-00146-x

[ref72] Pajares F. (2009). “Motivational role of self-efficacy beliefs in self-regulated learning” in Motivation and self-regulated learning: Theory, research, and applications. eds. Schunk D. H. Zimmerman B. J. (New York: Routledge), 111–139.

[ref73] Panadero E. Alonso-Tapia J. (2014). How do students self-regulate? Review of Zimmerman’s cyclical model of self-regulated learning. Anal. Psicol. 30, 450–462. doi: 10.6018/analesps.30.2.167221

[ref74] Paris S. G. Paris A. H. (2001). Classroom applications of research on self- regulated learning. Educ. Psychol. 36, 89–101. doi: 10.1207/S15326985EP3602

[ref75] Pellegrino J. Hilton M. (2012). Education for life and work: Developing transferable knowledge and skills in the 21st century. Washington, DC: National Academies Press.

[ref9003] Pintrich P. R. (2004). A conceptual framework for assessing motivation and self-regulated learning in college students. Educ. Psychol. Rev. 16, 385–407. doi: 10.1007/s10648-004-0006-x

[ref76] Pokhrel S. Chhetri R. (2021). A literature review on impact of COVID-19 pandemic on teaching and learning. Higher Educ. Future 8, 133–141. doi: 10.1177/2347631120983481

[ref77] Prensky M. (2005). In digital games for education, complexity matters. Educ. Technol. 45, 22–28.

[ref78] Samiei F. Ebadi S. (2021). Exploring EFL learners’ inferential reading comprehension skills through a flipped classroom. Res. Pract. Technol. Enhanc. Learn. 16:12. doi: 10.1186/s41039-021-00157-9

[ref79] Shih H. C. J. Huang S. H. C. (2020). College students’ metacognitive strategy use in an EFL flipped classroom. Comput. Assist. Lang. Learn. 33, 755–784. doi: 10.1080/09588221.2019.1590420

[ref80] Shin D. S. Cimasko T. Yi Y. (2020). Development of metalanguage for multimodal composing: a case study of an L2 writer’s design of multimedia texts. J. Second. Lang. Writ. 47:100714. doi: 10.1016/j.jslw.2020.100714

[ref81] Slavin R. E. (1991). Synthesis of research of cooperative learning. Educ. Leadersh. 48, 71–82.

[ref82] Sommer M. Ritzhaupt A. (2018). Impact of the flipped classroom on learner achievement and satisfaction in an undergraduate technology literacy course. J. Inf. Technol. Educ. Res. 17, 159–182. doi: 10.28945/4059

[ref83] Stockwell G. (2013). “Tracking learner usage of mobile phones for language learning outside of the classroom” in Learner-computer interaction in language education: A festschrift in honor of Robert Fischer. CALICO monograph series. eds. Hubbard P. Schulze M. Smith B. (CALICO: San Marcos, TX), 118–136.

[ref84] Stöhr C. Demazière C. Adawi T. (2020). The polarizing effect of the online flipped classroom. Comput. Educ. 147:103789. doi: 10.1016/j.compedu.2019.103789

[ref85] Strayer J. F. (2012). How learning in an inverted classroom influences cooperation, innovation and task orientation. Learn. Environ. Res. 15, 171–193. doi: 10.1007/s10984-012-9108-4

[ref86] Strelan P. Osborn A. Palmer E. (2020). The flipped classroom: a meta-analysis of effects on student performance across disciplines and education levels. Educ. Res. Rev. 30:100314. doi: 10.1016/j.edurev.2020.100314

[ref88] Subedi S. Nayaju S. Subedi S. Shah S. K. Shah J. M. (2020). Impact of E-learning during COVID-19 pandemic among nursing students and teachers of Nepal. Int. J. Sci. Healthc. Res. 5, 68–76.

[ref87] Su Ping R. L. Verezub E. Adi Badiozaman I. F. B. Chen W. S. (2020). Tracing EFL students’ flipped classroom journey in a writing class: lessons from Malaysia. Innov. Educ. Teach. Int. 57, 305–316. doi: 10.1080/14703297.2019.1574597

[ref90] Sweeny S. M. (2010). Writing for the instant messaging and text messaging generation: using new literacies to support writing instruction. J. Adolesc. Adult. Lit. 54, 121–130. doi: 10.1598/JAAL.54.2.4

[ref91] Syatriana E. (2011). *Developing the students’ reading comprehension through cognitive reading strategies of the first year students of SMAN 16 Makassar (unpublished master's thesis)*. Indonesia.

[ref92] Tan C. Yue W.-G. Fu Y. (2017). Effectiveness of flipped classrooms in nursing education: systematic review and meta-analysis. Chin. Nurs. Res. 4, 192–200. doi: 10.1016/J.CNRE.2017.10.006

[ref93] Tang T. Abuhmaid A. M. Olaimat M. Oudat D. M. Aldhaeebi M. Bamanger E. (2023). Efficiency of flipped classroom with online-based teaching under COVID-19. Interact. Learn. Environ. 31, 1077–1088. doi: 10.1080/10494820.2020.1817761

[ref94] Theobald M. (2021). Self-regulated learning training programs enhance university students’ academic performance, self-regulated learning strategies, and motivation: a meta-analysis. Contemp. Educ. Psychol. 66:101976. doi: 10.1016/j.cedpsych.2021.101976

[ref95] Topping K. Ehly S. (1998). Peer assisted learning. Oxford: Routledge.

[ref96] Tse S. K. Lin L. Ng R. H. W. (2022). Self-regulated learning strategies and reading comprehension among bilingual primary school students in Hong Kong. Int. J. Biling. Educ. Biling. 25, 3258–3273. doi: 10.1080/13670050.2022.2049686

[ref97] Turan Z. Akdag-Cimen B. (2020). Flipped classroom in English language teaching: a systematic review. Comput. Assist. Lang. Learn. 33, 590–606. doi: 10.1080/09588221.2019.1584117

[ref98] University of Cambridge ESOL Examinations. (2011). *Score processing, reporting and interpretation*. http://www.ielts.org/researchers/score_processing_and_reporting.aspx. (Accessed November 11, 2011).

[ref99] Van Alten D. C. Phielix C. Janssen J. Kester L. (2020). Self-regulated learning support in flipped learning videos enhances learning outcomes. Comput. Educ. 158:104000. doi: 10.1016/j.compedu.2020.104000

[ref100] Van Laer S. Elen J. (2017). In search of attributes that support self-regulation in blended learning environments. Educ. Inf. Technol. 22, 1395–1454. doi: 10.1007/s10639-016-9505-x

[ref101] Vitta J. P. Al-Hoorie A. H. (2020). The flipped classroom in second language learning: a meta-analysis. Lang. Teach. Res. 27:1403. doi: 10.1177/1362168820981403

[ref102] Wanner T. Palmer E. (2015). Personalising learning: exploring student and teacher perceptions about flexible learning and assessment in a flipped university course. Comput. Educ. 88, 354–369. doi: 10.1016/j.compedu.2015.07.008

[ref103] Weir C. J. O’Sullivan B. (2017). Assessing English on the global stage: The British Council and English language testing, 1941—2016. Mumbai: Equinox.

[ref104] Widayanti R. Meria L. (2023). Business modeling innovation using artificial intelligence technology. Int. Trans. Educ. Technol. 1, 95–104. doi: 10.33050/itee.v1i2.270

[ref105] Woodford P. E. (1982). *An introduction to TOEIC: The initial validity study*. Princeton, NJ: Educational Testing Service. Available at: https://www.ets.org/Media/Research/pdf/TOEIC-RS-00.pdf.

[ref106] Wu Y. A. (2001). English language teaching in China: trends and challenges. TESOL Q. 35, 191–194. doi: 10.2307/3587867

[ref107] Yang X. Kuo L. J. Eslami Z. R. Moody S. M. (2021). Theoretical trends of research on technology and L2 vocabulary learning: a systematic review. J. Comput. Educ. 8, 465–483. doi: 10.1007/s40692-021-00187-8

[ref108] Yapp D. J. de Graaff R. van den Bergh H. (2021). Improving second language reading comprehension through reading strategies: a meta-analysis of L2 reading strategy interventions. J. Sec. Lang. Stud. 4, 154–192. doi: 10.1075/jsls.19013.yap

[ref109] Yulian R. (2021). The flipped classroom: improving critical thinking for critical reading of EFL learners in higher education. Stud. Engl. Lang. Educ. 8, 508–522. doi: 10.24815/siele.v8i2.18366

[ref110] Zainuddin Z. Attaran M. (2016). Malaysian students’ perceptions of flipped classroom: a case study. Innov. Educ. Teach. Int. 53, 660–670. doi: 10.1080/14703297.2015.1102079

[ref111] Zarifis A. Efthymiou L. (2022). *The four business models for AI adoption in education: giving leaders a destination for the digital transformation journey*. In *2022 IEEE global engineering education conference (EDUCON)*. IEEE, pp. 1868–1872.

[ref112] Zarrinabadi N. Ebrahimi A. (2018). Increasing peer collaborative dialogue using a flipped classroom strategy. Innov. Lang. Learn. Teach. 13, 267–276. doi: 10.1080/17501229.2018.1455688

[ref113] Zhang S. Zhang X. (2022). The relationship between vocabulary knowledge and L2 reading/listening comprehension: a meta-analysis. Lang. Teach. Res. 26, 696–725. doi: 10.1177/1362168820913998

[ref115] Zimmerman B. J. (2002). Becoming a self-regulated learner: an overview. Theory Pract. 41, 64–70. doi: 10.1207/s15430421tip4102_2

[ref114] Zimmerman B. J. (2000). “Attaining self-regulation: a social cognitive perspective” in Handbook of self-regulation. eds. Boekaerts M. Pintrich P. R. Zeidner M. (San Diego, CA: Academic Press), 13–39.

[ref116] Zimmerman B. J. Moylan A. R. (2009). “Self-regulation: where metacognition and motivation intersect” in Handbook of metacognition in education. eds. Hacker D. J. Dunlosky J. Graesser A. C. (New York: Routledge), 299–315.

[ref117] Zimmerman B. J. Schunk D. H. (2001). Self-regulated learning and academic achievement: Theoretical perspectives. Abingdon: Routledge.

